# Improved Non-Negative Matrix Factorization-Based Noise Reduction of Leakage Acoustic Signals

**DOI:** 10.3390/s24165146

**Published:** 2024-08-09

**Authors:** Yongsheng Yu, Yongwen Hu, Yingming Wang, Zhuoran Cai

**Affiliations:** 1 State Key Laboratory of Silicate Materials for Architecture, Wuhan University of Technology, Wuhan 430070, China; 2 School of Naval Architecture, Ocean and Energy Power Engineering, Wuhan University of Technology, Wuhan 430070, China; huyw3366@hotmail.com (Y.H.); ymingwang@whut.edu.cn (Y.W.); czr@whut.edu.cn (Z.C.)

**Keywords:** non-negative matrix factorization (NMF), KL scatter, noise reduction algorithms, acoustic signal analysis, adaptive MMSE-LSA method, signal-to-noise ratios

## Abstract

The detection of gas leaks using acoustic signals is often compromised by environmental noise, which significantly impacts the accuracy of subsequent leak identification. Current noise reduction algorithms based on non-negative matrix factorization (NMF) typically utilize the Euclidean distance as their objective function, which can exacerbate noise anomalies. Moreover, these algorithms predominantly rely on simple techniques like Wiener filtering to estimate the amplitude spectrum of pure signals. This approach, however, falls short in accurately estimating the amplitude spectrum of non-stationary signals. Consequently, this paper proposes an improved non-negative matrix factorization (INMF) noise reduction algorithm that enhances the traditional NMF by refining both the objective function and the amplitude spectrum estimation process for reconstructed signals. The improved algorithm replaces the conventional Euclidean distance with the Kullback–Leibler (KL) divergence and incorporates noise and sparse constraint terms into the objective function to mitigate the adverse effects of signal amplification. Unlike traditional methods such as Wiener filtering, the proposed algorithm employs an adaptive Minimum Mean-Square Error-Log Spectral Amplitude (MMSE-LSA) method to estimate the amplitude spectrum of non-stationary signals adaptively across varying signal-to-noise ratios. Comparative experiments demonstrate that the INMF algorithm significantly outperforms existing methods in denoising leakage acoustic signals.

## 1. Introduction

Different gases stored and transported in pressure vessels or pipelines are integral to modern life and industry. These gases range from natural gas used for heating and cooking to industrial gases like oxygen and nitrogen essential for various manufacturing processes. As the equipment ages and third-party damage occurs, gas leaks have become increasingly prominent, posing risks of environmental pollution and threats to property and personal safety. A common culprit is sulfur hexafluoride (SF_6_), used extensively in electrical substation transformers due to its excellent insulating properties. However, leaks of SF_6_ compromise the safety performance of electrical equipment and contribute to air pollution. This potent greenhouse gas has a global warming potential significantly higher than CO_2_, making its release into the atmosphere particularly concerning. Thus, effective gas leak detection is crucial for mitigating such hazards.

Traditional gas leak detection methods are categorized into mechanism-driven and data-driven approaches. Mechanism-driven approaches involve constructing predictive models based on the gas’s physical properties, such as vacuum, laser imaging, and infrared detection methods [1,2,3]. These methods are often constrained by their low detection accuracy, high cost, and impracticality for real-time field testing. In contrast, data-driven methods significantly depend on the type of input signal—whether vibration, pressure, or acoustic signals—which greatly influences detection outcomes [4,5,6]. This dependency on signal type makes data-driven methods more adaptable to various environmental conditions and potentially more effective in complex scenarios. Notably, acoustic signal-based inspections offer non-contact measurement, crucial for ensuring staff health and safety during gas leak detection. This feature not only protects the personnel from potential hazards but also prevents any disturbance to the infrastructure during inspection. Acoustic signals also span a broad frequency range from infrasound to ultrasound, encapsulating extensive fault information. This wide range allows for the detection of minute anomalies in the system, thereby increasing the sensitivity of the detection process. However, collected acoustic signals frequently include background noise, complicating the classification and identification processes and thereby impacting leak detection accuracy. The presence of such noise can mask subtle leak signals, making early detection difficult and sometimes leading to false positives or negatives. Consequently, developing an effective noise reduction algorithm for leak sound is essential for enhancing detection reliability.

Acoustic signal noise reduction is a crucial challenge in the field of acoustics, which aims to separate clean target signals from noise-contaminated signals. Acoustic signal noise reduction methods can be divided into traditional noise reduction methods and machine learning-based noise reduction methods, reflecting the principle and development history of noise reduction. Traditional acoustic noise reduction methods are mainly based on digital signal processing techniques and include approaches such as spectral subtraction [7], the Wiener filtering method [8], the statistical modeling method [9], and the subspace method [10]. However, these methods often rely on certain assumptions or minimal use of a priori information of pure acoustic and noisy signals, which results in diminished effectiveness against non-stationary noise. Audio noise reduction can be considered a supervised learning problem, and an increasing number of experts and scholars are employing machine learning methods to enhance the effectiveness of audio noise reduction. Machine learning methods can be further divided into those based on traditional machine learning models [11] and those based on deep learning models [12]. Deep learning-based modeling methods require larger computational resources and sufficient acoustic signals to train an excellent noise reduction model. However, gas leakage represents a type of low-probability event, making it challenging to collect ample leakage data and noise data for training. Consequently, noise reduction methods based on traditional machine learning models, owing to their lesser requirements for computational resources and data volume, are more suitable for scenarios such as gas leak detection.

The non-negative matrix factorization (NMF)-based noise reduction algorithm is a commonly utilized machine learning noise reduction algorithm that acquires different signal bases of pure and noisy signals by training the noisy audio and pure audio separately and then using the different signal bases to separate the noisy signals. It efficiently utilizes the a priori information of the noise-containing signal to capture the amplitude spectrum of the pure signal and the noisy signal at the time of training. Subsequently, it can be weighted as a linear combination of basis vectors by modeling them as negative activation coefficients.

Since the NMF-based noise reduction algorithm requires pure signals and noisy signals as a priori knowledge for training, the algorithm performance will be dramatically degraded when the disparity between the training data and the noisy signals is substantial and the trained basis matrix cannot effectively represent the noisy signals. Consequently, Zhang Lijun et al. [13] proposed an RNMF algorithm by incorporating a noise constraint term into the objective function to address the random errors generated during the decomposition of non-negative matrices. He Wei et al. [14] introduced an SRNMF algorithm by adding a sparse regularization term to the coefficient matrix in the objective function to manage the sparsity. However, both approaches utilize the Euclidean distance as the objective function to measure the error before decomposition and after reconstruction. For the high-energy amplitude of the background noise signal, it is prone to cause the noise to be amplified and compromise the algorithm’s performance. Moreover, the prevailing NMF-based noise reduction algorithms primarily employ Wiener filtering and other basic estimations of the amplitude spectrum of the signal, leading to issues such as inaccurate estimation of the changing non-stationary noise and inability to adaptively estimate the amplitude spectrum.

To address the above problems, this paper proposes an improved non-negative matrix factorization (INMF) noise reduction algorithm. The proposed INMF-based noise reduction method takes Kullback–Leibler (KL) divergence as the objective function and introduces noise terms and sparse constraint terms in the objective function, which is superior to the ones with Euclidean distance. Moreover, an improved adaptive Minimum Mean-Square Error-Log Spectral Amplitude (MMSE-LSA) sub-algorithm is embedded to estimate the amplitude spectrum of the reconstructed signal, compared with the current NMF series noise reduction algorithm using simple estimation methods such as Wiener filtering. The experimental results show that the proposed INMF noise reduction algorithm has a better noise reduction effect on the leakage acoustic signal.

## 2. INMF-Based Noise Reduction Algorithm

### 2.1. INMF Algorithm

With the development of NMF, it has been extensively applied to and refined across various fields. Zhang Lijun et al. [13] and He Wei et al. [14] modified it by using Euclidean distance as the objective function; however, this method tends to exacerbate errors caused by anomalies. Grady et al. formulated the objective function under KL divergence dispersion by deriving the coefficient matrix H and the basis matrix W, assuming that the anomalous noise follows a Poisson distribution [15]. Nevertheless, when applying non-negative matrix decomposition to process acoustic signal data, the actual algorithm performance often deviates significantly from theoretical predictions due to the random nature of the errors that may arise. Consequently, the noise term E is introduced here to mitigate the impact of these issues, i.e., X≈WH+E. The interference of the coefficient matrix H and the basis matrix W by noise can be effectively reduced by the introduced noise term, and the objective function is expressed as Equation (1):

(1) $$D_{KL}\left(X,\hat{X},E\right)=\sum_{k=1}^{K}\;\sum_{l=1}^{L}\;\left[(x-e{)}_{k,l}lg\frac{(x-e{)}_{k,l}}{{\hat{x}}_{k,l}}-(x-e{)}_{k,l}+{\hat{x}}_{k,l}\right]$$

where $D_{KL}(X,\hat{X},E)$ is the objective function under KL divergence, X is the amplitude spectrum of the noisy signal, $\hat{X}$ is the amplitude spectrum of the noisy air leakage signal after noise reduction, E is the introduced noise term, K is the frequency point of the noisy air leakage signal, L is the time frame of the noisy air leakage signal, x∈X, e∈E, and lg is a standard notation for the logarithm.

In order to enhance the sparsity of Equation (1), we consider adding a parametric constraint term to the noise term. Since the solution of $L_{0}$ parametrization is difficult, $L_{1}$ parametrization is added as the noise term. Therefore, the objective function of optimization is Equation (2).

(2) $$\underset{W,H,E}{min}\;D_{KL}\left(X,\hat{X},E\right)+\lambda ∥E{∥}_{1}\;s.\;t.\;W⩾0,H⩾0$$

where $∥E{∥}_{1}=\sum_{i=1}^{K}\;\sum_{l=1}^{L}\;\left|e_{k,l}\right|$ and λ is the trade-off coefficient that controls the weight of E sparsity and reconstruction error, which can control the weight of E.

Given the sparse nature of time and frequency domains in the acoustic signal, the sparsity factor is crucial in determining the sparsity of the coefficient matrix H, thereby enabling the basis matrix W to serve as a perfect basis [16]. Consequently, the sparsity constraint term for the coefficient matrix H is proposed to be included in Equation (2) to enhance control over the sparsity of H and the distortion of the audio signal, thus refining the objective function to become Equation (3).

(3) $$\sum_{k=1}^{K}\;\sum_{l=1}^{L}\;\left[(x-e{)}_{k,l}lg\frac{(x-e{)}_{k,l}}{{\hat{x}}_{k,l}}-(x-e{)}_{k,l}+{\hat{x}}_{k,l}\right]+\lambda ∥E{∥}_{1}+\gamma ∥H{∥}_{1}$$

where γ ≥ 0 is the sparsity factor, which can control the sparsity of the coefficient matrix.

When the threshold operator is introduced, the convex optimization problem at update time can be solved by fixing W and H to continuously update the noise term added by the optimization [17]. Letting it be unbounded by the noise outlier form can improve the robustness of the algorithm. The soft threshold function ${soft}_{\lambda }(\cdot )$ can be expressed as Equation (4).

(4) $${soft}_{\lambda }(x)=\left\{\begin{array}{c} x-\lambda ,\;\;\;\;x>\lambda \\ x+\lambda ,\;\;\;\;x<-\lambda \\ 0,\;\;\;\;Other\; \end{array}\right.$$

where x∈R and λ>0 are used as thresholds. Equation (4) can be applied to matrices and vectors.

Given that there is no unique optimal solution for the objective function, the values of W, H, and E are established as Equations (5)–(7) by normalizing W and H, maintaining constant objective function values, and then optimizing Equation (3) via the gradient descent method.

(5) $$W\leftarrow W\circ \left(\frac{\left(X-E\right)\circ H^{T}}{1_{K\times L}\circ H^{T}}\right)$$

(6) $$H\leftarrow H\circ \left(\frac{W^{T}\circ \left(X-E\right)}{W^{T}\circ 1_{K\times L}+\gamma }\right)$$

(7) $$E\leftarrow {soft}_{\lambda }\left(X-WH\right)$$

where ∘ denotes the multiplication in the decomposition matrix, T denotes the transposition process, and $1_{K\times L}\in R^{K\times L}$ denotes the matrix with all element values of 1.

### 2.2. Improved Adaptive MMSE-LSA Algorithm

The MMSE-LSA algorithm is a method for joint estimation using a priori and a posteriori signal-to-noise ratios. In this paper, the value estimated for the acoustic signal differs because the acoustic signal is non-stationary. Benjebbour et al. determine the a priori S/N ratio by introducing a conditioning factor $\alpha^{\prime }$, which defines a fixed range for $\alpha^{\prime }$ and secures a more accurate empirical value by conducting several experiments [18]. However, this experimental approach to ascertaining the conditioning factor is not ideally suited for situations with varying audio signal-to-noise ratios. In contrast, the adaptive MMSE-LSA algorithm modifies the optimal value of the adjustment factor $\alpha^{\prime }$ at different stages of non-stationary noise in response to the variation in non-stationary noise, thus achieving enhanced results.

The MMSE-LSA algorithm obtains the estimated value of the denoised audio by minimizing Equation (8). |X(n,k)| can be expressed as Equation (9), and the estimate of the pure signal can be expressed as Equation (10).

(8) $$E\{(ln|X(n,k)|-ln|\hat{X}\left(n,k)|{)}^{2}\right\}$$

(9) |X(n,k)|=G(n,k)·|Y(n,k)|

(10) $$G(n,k)=\frac{\xi (n,k)}{\xi (n,k)+1}exp\left(\frac{1}{2}\int_{k}^{\infty }\;\frac{e^{-t}}{t}dt\right)$$

In Equation (9), G(n,k) is the estimated value of the air leakage signal after noise reduction. In Equation (10), ξ(n,k) is the a priori signal-to-noise ratio of the nth frame in the audio signal at the kth frequency point, and the signal-to-noise ratio is defined in Equation (11).

(11) $$\xi (n,k)=\frac{E\left\{|X(n,k){|}^{2}\right\}}{\lambda_{d}^{2}(n,k)}$$

From Equation (10), it can be seen that the a priori signal-to-noise ratio must be found first in order to estimate the pure audio signal. Therefore, the a priori signal-to-noise ratio has a direct impact on the audio noise reduction effect. The estimation of the a priori signal-to-noise ratio can be expressed as Equation (12).

(12) $$\hat{\xi }(n,k)=\alpha \cdot \frac{|\hat{X}(n-1,k){|}^{2}}{\lambda_{d}^{2}(n-1,k)}+(1-\alpha )P[\gamma (n,k)-1]$$

In Equation (12), $\left|\hat{X}(n-1,k)\right|$ is the pure signal spectral amplitude estimated at the kth component of the n−1 frame, $\lambda_{d}^{2}(n-1,k)$ is the estimated noise signal amplitude spectrum of the n−1 frame at the kth component, and α is the weighting factor.

(13) $$P[x]=\left\{\begin{array}{c} x\;\;\;\;x⩾0\\ 0\;\;\;\;x<0 \end{array}\right.$$

Equation (13) represents the half-wave rectification function, which is estimated using the minimum mean-square error (MMSE) in order to make the difference between $\hat{\xi }(n,k)$ and ξ(n,k) as small as possible.

(14) $$G=E\left\{\left(\hat{\xi }(n,k)-\xi (n,k){)}^{2}|\hat{\xi }(n-1,k)\right|\right\}$$

Substituting Equation (12) into Equation (14), Equation (15) is obtained.

(15) $$G=\alpha^{2}(n,k)(\hat{\xi }(n-1,k)-\xi (n-1,k){)}^{2}+(1-\alpha (n,k){)}^{2}\cdot (\xi (n,k)+1{)}^{2}$$

Find the derivative of G and let the partial derivative of G be 0. At this point, the optimal solution of α is obtained as Equation (16).

(16) $$\alpha_{opt}=\frac{1}{1+{\left(\frac{\xi (n,k)-\xi (n-1,k)}{\xi (n,k)+1}\right)}^{2}}$$

In Equation (16), $\bar{\xi }(n,k)=P\{\xi (n,k)-1\}$ is adopted, instead of the unknown ξ(n,k), for $E\{\bar{\xi }(n,k)\}≅\xi (n,k)$. The value of α(n,k) converges to 1 when the a posteriori signal-to-noise ratio changes, and $\hat{\xi }(n,k)$ changes when the value of α(n,k) is small, thus achieving an adaptive effect.

### 2.3. Principle of INMF Noise Reduction Algorithm

**Figure 1:**
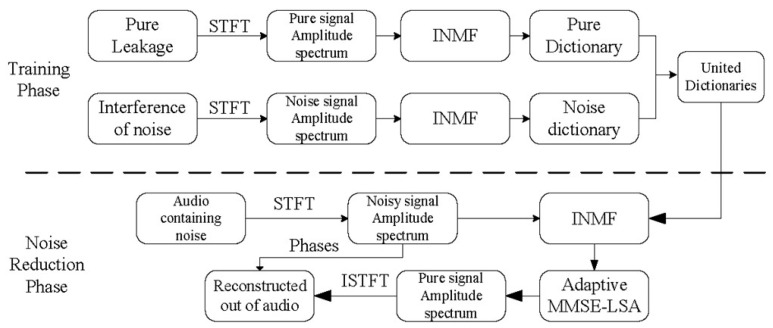
Flowchart of the INMF-based noise reduction.

The flowchart of the noise reduction is shown in Figure 1. The proposed INMF algorithm in this paper is applied to the noise reduction of acoustic signals in two main steps: one is the supervised learning training process, and the other is the audio noise reduction process, as shown in Figure 1.

**Figure 2:**
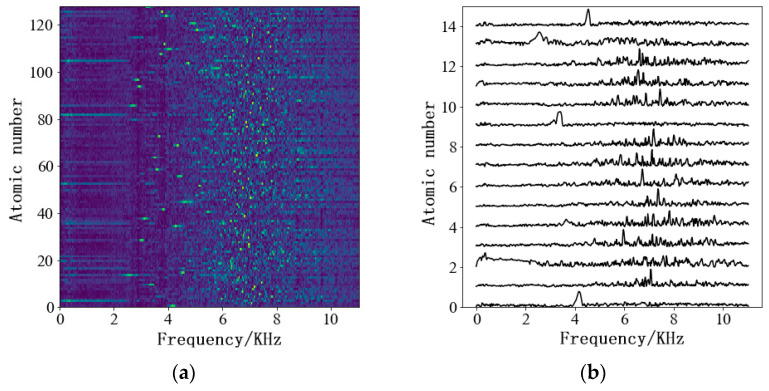
INMF learning dictionary for air leakage audio signal. (**a**) Dictionary matrix. (**b**) Dictionary atoms.

In the training process, the information of the pure air leakage signal and the interference noise signal are each captured using the short-time Fourier transform (STFT). Subsequently, the amplitude spectra of the pure air leakage signal and the interference noise signal are used as the target matrices, with $V_{L}\;$≥ 0 and $V_{N}\;$≥ 0. Following this, the aforementioned INMF algorithm is employed to iterate through Equations (5)–(7), with the number of iterations established at 100 to decompose the amplitude spectrum of the air leakage signal into the $W_{L}$ dictionary matrix and the amplitude spectrum of the interference noise into the $W_{N}$ dictionary matrix. The dictionary matrix of the air leakage signal is depicted in Figure 2a, where the columns of the dictionary matrix are termed dictionary atoms, as illustrated in Figure 2b. These dictionary atoms are non-negative functions of the frequency and can be linearly combined with the corresponding coefficients in real time. The decomposed $W_{L}$ and $W_{N}$ are combined to form a joint dictionary matrix $W=\left[\begin{array}{c} W_{L}\;\;\;\;W_{N} \end{array}\right]$, utilizing the joint dictionary as a priori information for the training process.

In the audio noise reduction process, the amplitude spectrum of the signal containing the interference noise is first obtained using the short-time Fourier transform, and the joint dictionary matrix W obtained using this amplitude spectrum and the training process is used as the input parameter of Equation (6); then, W is kept constant and updated iteratively using Equations (6) and (7) until the objective function reaches convergence and terminates. At the end of the iteration, the amplitude spectra of the pure air leakage audio signal ${\hat{V}}_{L}$ and the interference noise signal ${\hat{V}}_{N}$ are estimated and can be derived as Equation (17).

(17) $$X\approx WH=\left[\begin{array}{c} W_{L}\;\;\;\;W_{N} \end{array}\right]{\left[\begin{array}{c} H_{L}\;\;\;\;H_{N} \end{array}\right]}^{T}={\hat{V}}_{L}+{\hat{V}}_{N}=W_{L}H_{L}+W_{N}H_{N}$$

At the end of the iterative update iteration, the improved adaptive MMSE-LSA algorithm described above is used as the gain function $\tilde{G}$, i.e.,

(18) $$\tilde{G}=\left(W_{L}H_{L}\right)\cdot /\left(W_{L}H_{L}+W_{N}H_{N}\right)$$

The pure air leakage audio amplitude spectrum, denoted as ${\tilde{X}}_{L}=\tilde{G}*X$, can be estimated in real time. This approach overcomes the limitations of traditional methods such as Wiener filtering, which cannot effectively estimate signals that change in real time. Finally, by utilizing the invariance of the noise-containing frequency phase, the noise-reduced time-domain air leakage signal is obtained through the inverse short-time Fourier transform (ISTFT).

## 3. Experiments and Analysis of Results

### 3.1. Experimental Parameter Setting

The Source-to-Distortion Ratio (SDR) quantifies the overall quality of both air leakage signal distortion and noise reduction, which is frequently used to evaluate the performance of different noise reduction algorithms. SDR is measured in dB, and a higher value of SDR indicates less residual noise and a more pronounced noise reduction effect [19]. The Perceptual Evaluation of Speech Quality (PESQ) is an evaluation metric widely used to assess the performance of noise reduction, and a higher PESQ value signifies enhanced signal quality after noise reduction [20].

In the experiments conducted at Wuhan University of Technology, the pure gas leakage audio was sourced from recordings made in a semi-anechoic chamber using simulated gas leakages, alongside typical disturbance noises from a field substation environment, including wind, rain, birdsong, and cicada chirping. The sampling frequency was set at 44.1 kHz. The complete leakage process was captured, yielding 40 segments of 15 s audio clips for both the gas leak and each type of noise used for training. The Wavfile and Soundfile libraries facilitated the mixing of pure air leakage data with noise data, synthesizing noisy air leakage data at signal-to-noise ratios of −10 dB, −5 dB, 0 dB, 5 dB, and 10 dB. The noise reduction algorithm employed a Hamming window function with a frame length of 512 points, a frame shift of 128 points, and a maximum of 100 iterations.

### 3.2. Experimental Results and Analysis

**Figure 3:**
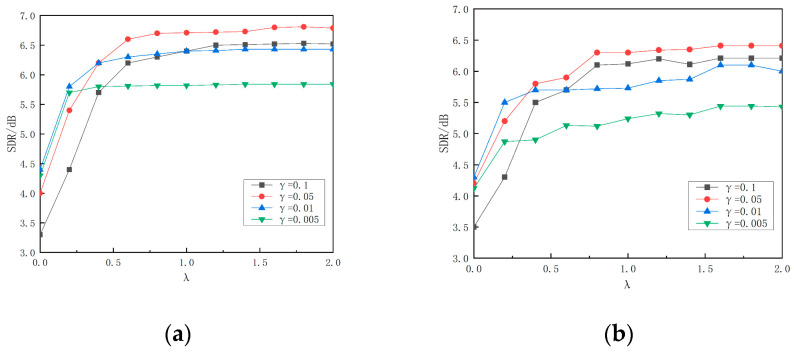

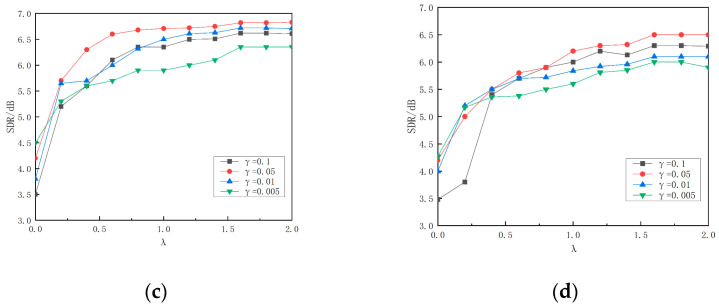
Average SDR values of noise reduction under different noises when the input signal-to-noise ratio is 0 dB. (**a**) Wind sound signal. (**b**) Rain signal. (**c**) Birdsong signal. (**d**) Cicada sound signal.

To ascertain the values of the INMF trade-off factor λ and the sparsity factor γ, experiments are conducted using noise-containing air leakage data. The sum of the SDR assesses the impact of λ and γ on noise reduction. Figure 3 illustrates how λ and γ influence the source distortion rate at an input signal-to-noise ratio of 0 dB.

As illustrated in Figure 3, the average SDR of the source distortion rate gradually increases before stabilizing under various background noise conditions with an increase in the trade-off coefficient λ. The value of λ significantly influences the performance of the improved non-negative matrix decomposition. After a comprehensive analysis, optimal parameters were identified at λ=1.6 and γ = 0.05. Consequently, to further evaluate the performance of the enhanced noise reduction algorithm, subsequent experiments utilized these parameters.

To evaluate the effectiveness of the INMF objective function and the adaptive LSA-MMSE estimation of the magnitude spectrum within the proposed INMF noise reduction algorithm, several comparisons were conducted. The INMF algorithm was utilized as the objective function, alongside comparisons with the traditional Wiener filter for amplitude spectrum estimation (termed Half INMF (HINMF)), the VMD algorithm (which is noted for its efficacy in denoising non-stationary noise [21]), the RNMF algorithm [13], and the SRNMF algorithm [14].

Table 1 and Table 2 display the comparison of SDR and PESQ values across four typical background noises at varying signal-to-noise ratios. The performance metrics derived from different noise reduction algorithms vary considerably. At high signal-to-noise ratios, VMD is more effective than RNMF in reducing wind noise but less effective compared to SRNMF, HINMF, and INMF, which offer deeper improvements. Specifically, VMD performs suboptimally against fluctuating non-stationary noises such as birdsong compared to the refined NMF-based algorithms, which excel by effectively leveraging training samples and enhancing noise reduction in non-stationary environments. Among the four NMF algorithms—RNMF, SRNMF, HINMF, and INMF—HINMF incorporates noise terms and sparse constraints in its objective function, significantly improving the preservation of acoustic characteristics and reducing performance degradation from anomalies. Conversely, INMF, which builds upon HINMF by adding adaptive MMSE-LSA for real-time amplitude spectrum estimation, further enhances algorithm robustness and noise reduction effectiveness. As signal-to-noise ratios increase, the relative advantage of INMF over other algorithms diminishes, yet it maintains a commendable noise reduction capability. Overall, INMF demonstrates superior noise reduction at low signal-to-noise ratios, with a reduced but still notable advantage at high ratios.

**Figure 4:**
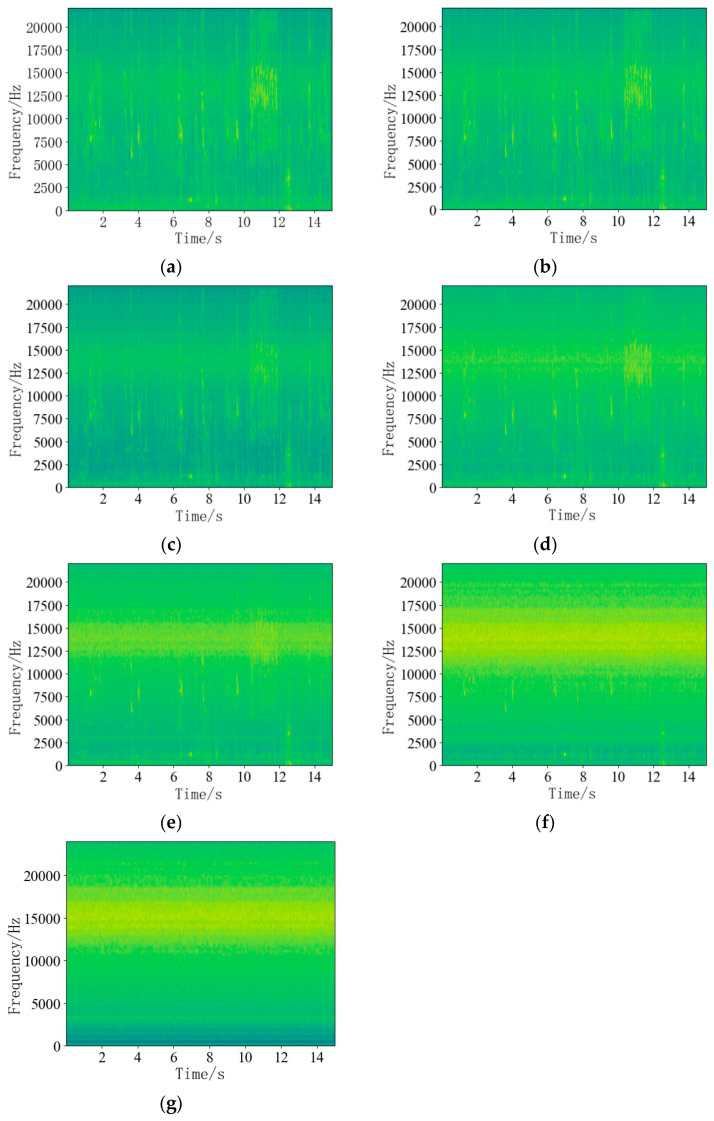
Time and frequency diagram of noise reduction of each algorithm with bird noise at 0 dB input signal-to-noise ratio. (**a**) Noisy signal. (**b**) VMD. (**c**) RNMF. (**d**) SRNMF. (**e**) HINMF. (**f**) INMF. (**g**) Noiseless signal.

To visually illustrate the differential effects of each algorithm on noise reduction, a time–frequency diagram is employed to showcase the noise reduction performance of each algorithm at a signal-to-noise ratio of 0 dB and under typical non-stationary noise conditions such as birdsong. The results are shown in Figure 4.

An analysis of Figure 4 reveals that the target signal is a broadband signal contaminated by typical non-stationary noise, which is irregularly distributed across the broadband spectrum of the air leakage signal. A comparative analysis indicates that VMD and RNMF exhibit substantial residual noise, suggesting weaker denoising capabilities. Conversely, SRNMF, HINMF, and INMF demonstrate stronger removal of non-stationary noises like birdsong, with INMF showing the least residual noise and the most effective noise reduction.

## 4. Discussion

This study focuses on the development and evaluation of an improved non-negative matrix factorization (INMF) algorithm for noise reduction in leakage acoustic signals. While the proposed method demonstrates superior performance in reducing non-stationary noise, it has several limitations. Firstly, the algorithm requires a higher computational cost due to its complexity, which may not be suitable for real-time applications with limited processing power. Secondly, the algorithm’s effectiveness in various real-world scenarios needs further validation, as the current study primarily uses synthetic noise data for evaluation. Finally, the performance of the proposed method was evaluated using synthetic noise data, which may not fully represent the variability and complexity of real-world noise environments. Future work should include comprehensive testing with real-world data and a thorough analysis of computational costs.

## 5. Conclusions

An INMF-based noise reduction algorithm for leakage acoustic signals is proposed in this paper, which addresses the problems of the current NMF-based noise reduction algorithms and related variants. The algorithm enhances the objective function of traditional non-negative matrix decomposition by incorporating noise terms and sparse constraint terms, thus mitigating the amplification of noise anomalies during decomposition. Optimal trade-off coefficients and sparse factors are determined through experimentation. Additionally, an adaptive MMSE-LSA method is introduced for estimating the amplitude spectrum, which adjusts to variations in non-stationary noise across different signal-to-noise ratios, enabling adaptive estimation of the amplitude spectrum. Comparative experiments confirm that the INMF algorithm offers superior denoising performance across various environmental noises and signal-to-noise ratios.

## Figures and Tables

**Table 1:** Output SDR/dB under different noise environments.

Input SNR/dB	Noise	VMD	RNMF	SRNMF	HINMF	INMF
−10	Wind	−4.13	−4.27	−3.28	−1.47	−1.44
	Rain	−3.62	−2.97	−2.62	−2.32	−2.02
	Bird	−4.14	−2.78	−2.66	−1.40	−1.32
	Cicada	−4.55	−4.25	−3.90	−2.33	−1.88
−5	Wind	0.79	0.75	1.25	1.24	1.23
	Rain	−1.15	−0.75	0.43	0.55	0.52
	Bird	−0.05	0.25	0.89	0.77	1.02
	Cicada	−1.77	−0.96	0.37	0.35	0.40
0	Wind	5.86	5.52	6.87	6.85	6.83
	Rain	4.5	5.06	6.43	6.45	6.49
	Bird	3.57	5.49	6.03	6.27	6.82
	Cicada	3.57	4.89	6.10	6.31	6.5
5	Wind	8.34	8.24	9.45	9.04	9.23
	Rain	8.15	8.20	8.35	8.35	8.46
	Bird	8.42	9.30	9.77	9.82	9.95
	Cicada	7.12	8.0	8.24	8.38	8.43
10	Wind	13.38	13.20	14.42	13.87	14.02
	Rain	11.24	10.38	11.01	12.05	12.43
	Bird	11.27	12.52	13.84	13.97	14.25
	Cicada	10.25	10.23	12.07	12.47	12.85

**Table 2:** Output PESQ/dB under different noise environments.

Input SNR/dB	Noise	VMD	RNMF	SRNMF	HINMF	INMF
−10	Wind	1.12	1.10	1.35	1.47	1.69
	Rain	1.07	1.09	1.21	1.39	1.45
	Bird	1.05	1.10	1.18	1.34	1.41
	Cicada	1.09	1.13	1.25	1.35	1.50
−5	Wind	1.43	1.41	1.63	1.79	1.77
	Rain	1.38	1.40	1.52	1.74	1.71
	Bird	1.34	1.39	1.48	1.65	1.72
	Cicada	1.37	1.41	1.54	1.67	0.40
0	Wind	1.73	1.69	1.97	2.09	2.08
	Rain	1.66	1.68	1.82	1.98	2.06
	Bird	1.65	1.72	1.77	1.99	2.04
	Cicada	1.68	1.73	1.64	1.69	2.05
5	Wind	2.02	2.37	2.63	2.73	2.96
	Rain	1.97	2.35	2.58	2.66	2.87
	Bird	1.95	2.28	2.55	2.60	2.88
	Cicada	1.96	2.30	2.54	2.63	2.85
10	Wind	2.23	2.58	2.82	2.91	3.08
	Rain	2.18	2.53	2.78	2.85	3.05
	Bird	2.15	2.49	2.75	2.84	3.01
	Cicada	2.20	2.52	2.74	2.86	3.02

## Data Availability

The data used to support the findings of this study are available from the corresponding author upon request.
